# A critical review and analysis of the context, current burden, and application of policy to improve cancer equity in Ghana

**DOI:** 10.1186/s12939-023-02067-2

**Published:** 2023-12-08

**Authors:** Chloe Zabrina Tuck, Richard Cooper, Richmond Aryeetey, Laura A Gray, Robert Akparibo

**Affiliations:** 1 School of Medicine and Population Health, University of Sheffield, 30 Regent Street, Sheffield, S1 4DA UK; 2 School of Public Health, University of Ghana, Accra, Ghana

**Keywords:** Cancer, Ghana, Non-communicable Diseases, Health policy, Health systems

## Abstract

**Background:**

Cancer causes a major disease burden worldwide. This is increasingly being realised in low and middle-income countries, which account disproportionately for preventable cancer deaths. Despite the World Health Organization calling for governments to develop policies to address this and alleviate cancer inequality, numerous challenges in executing effective cancer policies remain, which require consideration of the country-specific context. As this has not yet been considered in Ghana, the aim of this review was to bring together and critique the social-environmental, health policy and system factors to identifying opportunities for future health policies to reduce cancer burden in the Ghanian context. A critical policy-focused review was conducted to bring together and critique the current health systems context relating to cancer in Ghana, considering the unmet policy need, health system and social factors contributing to the burden and policy advances related to cancer.

**Conclusion:**

The findings highlight the changing burden of cancer in Ghana and the contextual factors within the socio-ecosystem that contribute to this. Policies around expanding access to and coverage of services, as well as the harmonization with medical pluralism have potential to improve outcomes and increase equity but their implementation and robust data to monitor their impact pose significant barriers.

## Background

Cancer causes an immense and growing burden on mortality worldwide. According to the 2022 GLOBOCAN report for cancer morbidity and mortality, the prevalence of cancers (all types) is estimated at about 19.3 million, and around 10 million deaths globally [1]. This burden is increasingly being felt in low- and middle-income countries who are undergoing an economic and epidemiological transition. Moreover, in Africa the share of global cancer incidence was 5.6% in 2020, yet the continent accounts for a greater share of the global cancer mortality, at 7.2% [1]. This highlights lower survival rates due to weaker health infrastructure to provide prevention and screening measures, as well as timely access to adequate treatment, and a need for policy advancement [1].

International recognition of the importance of cancer is illustrated in the influential 2020 ‘World Health Organization (WHO) report on cancer: s*etting priorities, investing wisely and providing care for all’* [2]; this covers more traditional prevention, screening and early diagnosis, cancer management and palliative care but also their related policy, financing, and the implementation of cancer plans [2]. It is recognised that the most effective policy approach will require tailoring to the national context. Although Ghana has a National Strategy for Cancer Control, launched in 2011 [3], significant hurdles still remain in achieving the aims of the plan. For example, cancer is increasingly a major burden in the Ghanaian context [4]. This policy review aims to examine the health system and policy environment and make suggestions that could be considered to address the unmet policy need to alleviate the burden of cancer in Ghana. Specific objectives for this review are to:

1. Analyse the burden of cancers, and implication on the health system.
2. Review social and health environment factors influencing increasing burden.
3. Review and critique government policies tackling cancers through an equity lens.

The findings of this will be combined to draw conclusions regarding any unmet policy need.

As a qualitative policy review literature, a purposive “compass-style” approach, guided by the relevance in accordance with emerging themes (rather than anchored predefined criteria) was taken [5]. Key governmental and international websites were first identified based on their relevance to cancer and Ghana, reviewed and a snowballing approach (purposively selecting reference and citations) was then applied. Literature within a 10-year period was included for policy relevance.

## What is the burden of cancer in Ghana?

Cancer contributes to the growing non-communicable disease burden in Ghana. The global cancer observatory estimate that there were 24 009 cancer cases and 15 802 cancer related deaths in Ghana in 2020 [6]. The majority of social epidemiological cancer literature in Ghana focuses on breast and cervical cancer [7], which may be justified given the major burdens they impose [4]. However, current trends in cancer rates and risk factors indicate there is a need to consider a broader range of cancers for future cancer service planning and policy development. Below, we have considered and discussed the common cancer types that pose a major health threat in Ghana.

Breast cancer remains the most common cancer in Ghana [1, 4] as it also is globally [1]. Lifestyle-associated risk factors are known to contribute to incidence rates in high income settings (including obesity, alcohol, smoking, later and reduced child-bearing), but now dramatic changes in the social-cultural environment in transitioning countries, such as an increase in women in the workforce, mean that these risk factors are increasing in low- and middle-income countries, including Ghana [1]. Late presentation in Ghana is associated with poor prognosis [8, 9]. Stigma around breast cancer means patients do not disclose their symptoms or seek treatment [10, 11]; those that do seek treatment often do not stay to complete the treatment, and so survival rates continue to be low [12]. High levels of defaulting (not completing treatment) are also linked to patients using traditional medicines [11–13] and being unable to afford treatment, [12, 14] which displays a social gradient [15].

Also affecting women and being linked to inequality, cervical cancer still represents a large burden in many low and middle income countries [1] including Ghana [4, 16], where it is the second most common cancer in females, according to local registry data and the 2022 GLOBOCAN estimates [6]. This is the case despite the global call to action to eliminate the burden of cervical cancer by the World Health Organization [17]. Cervical cancer is considered preventable with nationwide coverage of HPV vaccination and cervical cancer screening [16]. However, Ghana does not have a national HPV vaccination programme [18] and recommendations of the vaccination by health professionals is low due to poor availability and negative perceptions of the vaccine [19]. Screening rates are estimated as low as 16.9% in sub-Saharan Africa [1], and a study in Ghana found 97% of women have not been screened for cervical cancer and uptake was worse in low income and less educated groups [20]. Additionally, fatality rates are also higher in these contexts, due to late presentation and treatment, which exacerbates the burden [1].

Beyond the two cancer types discussed above, the burden of numerous other cancers and their associated risk factors, are becoming a public health concern in Ghana in recent years. For instance, prostate cancer, which remains the most common male cancer in Ghana according to local population registry data [4], (GLOBOCAN estimates 2129 cases per year in a population of 32 million in 2020 [6, 21]).

Liver cancer was the most common cause of cancer death in Ghana in 2020 according to the GLOBOCAN report (3166 deaths per year), and the second most common cancer for both sexes in terms of estimated cases (3452 cases per year) [1, 6]). These deaths are largely attributable to the late presentation of cases, which leads to a poor prognosis [22]. Most patients are only eligible for palliative care [23]. High liver cancer incidence may reflect high prevalence of risk factors including those related to poverty such as hepatitis B and C (HBV and HCV) infection [23–25]and aflatoxin exposure - as well as sedentary lifestyle [1]. In addition to prostate cancer and liver cancer, lung cancers may be of increasing concern in the Ghanaian setting if high rates of solid fuel use indoors continue. Solid fuel use is associated with cancer risk. In Ghana this is common and includes use of charcoal, wood and to a lesser extent, coconut shells in the costal areas. It is estimated to be as high as 85% in some populations [26]. This will disproportionately impact women, who cook more often in Ghana and the poor, who most frequently use such fuels. Lastly, colorectal cancer exerts a higher burden in high income than in low-income countries [27] but the burden is growing and spreading from rural to urban communities in Ghana where there is an increase in risk factors such as a sedentary lifestyle and excess body weight [1].

The burden of cancer is predicted to continue to increase globally, but most rapidly in low and middle income settings, [1, 28, 29] leading to increasing socio-economic inequality [28]. Of critical concern are the increasing lifestyle risk factors associated with cancer risk (sedentary lifestyle, excess weight, later and less childbearing) occurring in tandem with high levels of infection causing cancers such as Human Papilloma Virus (HPV) and HBV in Ghana [1]. This will inflict an even greater burden on health systems that may lack the capacity and financial resources to treat these cases [28]. There will therefore be a need to plan services to cater for the growing burden of cancer that can use the limited resource most efficiently. Equitable service planning, however, will also require an understanding of how to set priorities to address the cancer burden in Ghana and this, in turn, requires understanding the local social context, health architecture, and political environment which cancer is treated in, and these factors are now considered further.

## What contextual factors contribute to the cancer burden in Ghana?

The high proportion of preventable cancers in Ghana suggests there are multiple health system and social contextual factors that contribute to the disease burden, and in this section five factors are argued to be particularly relevant, linked to health service capacity and expertise, health service arrangements, medical pluralism, stigma and public cancer knowledge and awareness.

### Health service capacity and expertise

The Ghanaian health system has around 3,500 medical care centres which can be further divided into public, faith-based medical (operated by the Christian Health Association of Ghana (CHAG)) and private [30]. Public health facilities are funded by the Ministry of Health through government and donor partners. There are regional referral hospitals offering secondary care, district hospitals offering primary and emergency care, as well as community services (clinics, health centres, and community health planning and services/CHIPS compounds) [30, 31]. Community services have largely been established to prevent and treat communicable diseases, but do not have expertise in detecting non-communicable diseases such as cancer.

Cancer in Ghana is treated at major referral centres in the largest cities, Accra, Kumasi, and more recently Tamale and Cape Coast. It has been observed that most expertise in diagnosing cancer care resides at larger facilities such as regional and referral hospitals [32]. Community and district level services may not have sufficient expertise to recognise and refer suspected cancer patients.

Additionally, as most expert cancer care is exclusively in large cities [32], in poorresourced regions there is a lack of expertise due to insufficient training in oncology [33] as well as general gaps in human resource to provide cancer care [31]. A health workforce gap analysis found that the Ghana Health Service, which is the government health agency responsible for delivering public-funded/owned health care, was estimated to have over 47,000 vacancies in 2018 (a vacancy rate of 41%) with considerable variation in staffing between regions. Wellresourced regions were over twice as well staffed, with higher staffing in regional hospitals and urban centres [31]. In the peripheral, poor resourced regions and regional hospitals, there are also lower proportions of high cadre staff including clinicians and pharmacists. They estimate a net deficit in funding of 57% (approximately 300 million USD in 2021) to reach the minimum staff requirements [31]. However this could be reduced by 30% by redistributing staff more equitably [31].

### Health service arrangements

The health service set-up and patient’s referral process can also bring challenges, which can impact the care they receive and contribute to the cancer burden. The patient’s pathway can be considered holistically as a dynamic process whereby patients identify their need for, navigate, access and ultimately accept services [34]. The exact referral process will depend on the local context but routinely involves referral between departments and a series of diagnostic tests (either at the hospital or offsite), which may also incur additional costs. Our previous work highlighted multiple factors related to the referral process, such as having to go to multiple hospitals and laboratories, long wait times for laboratory results and consultant rescheduling, leading to delays in the patient pathway [7]. Moreover, inter-personal factors such as unequal power dynamics with clinical staff and poor communication about the treatment may impede the patient’s treatment decision making capacity [35].

### Medical pluralism

Alongside orthodox medical treatment at health facilities in Ghana, health and wellbeing support is also sought through several other services, often deemed as more affordable, available, accessible or accepted in community settings [36]. These include other medical providers: informal medicines sellers, drug/chemical shops, and pharmacies. It also extends to herbal medicines providers and practitioners offering non-medical services: traditional birth attendants, spiritual healers, bone-setters, Islamic-based healers and Christian prayer camps [37–39]. As well as their accessibility and affordability, traditional medicines are also perceived as natural (so safer) and efficacious [36]. These treatment options provide personalised treatment, assurance, comfort, and holistic wellbeing, which aligns with cultural values [36]. OThis is assuring and comforting and allowed patients to build a relationship with their provider [36]. This has been found to be most common in older and poorer people, those that are uninsured(on the NHIS), [40] and in rural areas with poor access to orthodox facilities [36]. This is likely associated with numerous intersecting characteristics related to age, education, poverty, and social exposures in rural and urban settings.

Research suggests patients seek multiple sources of care, in some instances these are sought alongside orthodox treatment [41, 42]. Patients switch between treatments and may seek traditional medicines as a first choice or after being dissatisfied with orthodox treatment [37, 42]. Studies have noted there may be scepticism of orthodox treatment as being foreign and not trusted [36]. Previous poor experiences [36, 37, 43] and negative interactions with health professionals pushes patients away [36]. Patients seldom report traditional medicines use to other health professionals [37, 44], bringing risk of drug interactions. This likely reflects adverse attitudes of health professionals towards plural treatments [38]. For example, one study in Northern Ghana found patients were insulted by healthcare professionals for using traditional medicines and the healers were not allowed into the health facilities [38].

This research highlights the need for collaboration between traditional and orthodox treatment in Ghana to holistically improve cancer care in a way that reflects the local context where patients may seek care from multiple sources.

### Health-related stigma

In addressing the burden of cancer in Ghana there is a need to consider cancer stigma, which has been highlighted in numerous studies to date [7]. This is also noted in the National Strategy for Cancer Control as an explicit barrier to treatment access in Ghana [3]. Stigma relates to beliefs, emotions, and patterns of behaviour, which infer disadvantage on a person based on a specific trait. This can be anticipated, perceived and internalised (felt or self-stigma) or experienced (enacted) [45, 46]. Stigma around cancer leads to secrecy, late diagnosis and poor treatment adherence [10, 14, 47]. This has been linked to perceptions of cancer being spiritual or a curse [48], being incurable, and the financial implications that it can drain families’ resources [49]. Indeed, breast cancer has been seen as a “death sentence” in Ghana [14]. However, for breast cancer in particular, recent campaigns, such as those centred around breast cancer awareness month, have attempted to increase awareness and acceptability to come forward to report breast cancer cases.

Stigma is also interlinked with societal gender roles, for women with breast cancer, mastectomy has been implicated with fears of diminished femininity [50]. Likewise, prostate cancer has been found to challenge masculine identities in men [51].

Stigma in Ghana is not unique to cancer but is seen in a multitude of other diseases. For example, stigma has been documented in communicable diseases such as Human Immunodeficiency Virus (HIV) [52–54], Tuberculosis [55], Covid-19 [56], Ebola [57] and Hepatitis [58], as well as non-communicable diseases such as sickle cell disease, [59] diabetes, [60] and mental illness [61]. Stigmatised conditions can be unified by their commonalities in dimensions: aesthetics, concealability, disease course, disruptiveness, origin (whether it is controllable), peril (whether it is curable) [62]. For example, many other stigmatised conditions (such as HIV) are, similarly to cancer, seen as incurable and caused by a personal choice.

Health-related stigmatisation leads to poor adherence, delayed treatment seeking and social marginalisation. This is common globally, and stigma has been shown to adversely impact health outcomes in low and middle income countries [45, 63]. Additionally, stigmatised identities are often intersectional and the impact is greater felt in groups already suffering other disadvantages [64]. Despite the widespread prevalence of stigma and the commonalities in stigma drivers and outcomes [62, 65], interventions and research are rarely cross cutting [45, 63, 66]. However, cross-cutting approaches could draw on a body of evidence and use evidence-based stigma tools, methods and interventions [66]. In overcoming cancer stigma in the Ghanaian context, there could be learning from interventions aimed at overcoming stigma for other conditions, such as HIV [48].

### Knowledge and awareness of cancer

Some of the most common cancers in Ghana could be preventable with early diagnosis and thus improved awareness of cancer symptoms and risks could improve outcomes [1, 4, 9, 16, 22]. However, for breast cancer [67] and cervical cancer [68] awareness of the symptoms has been found to be poor in some sub-populations, which often leads to late diagnosis. This is especially the case in less educated groups and more deprived or low-resourced regions [15, 67, 69]. There is often misinterpretation of symptoms as a minor condition or as spiritual in nature [70]. This extends to the understanding of health care professionals [71]. However, cancer awareness campaigns in recent years have sought to address this, with some improvements found in more recent studies [72, 73].

## What policies exist to tackle the cancer burden in Ghana?

This review so far has highlighted the cancer burden and factors that contribute to this within the social and healthcare environment in Ghana. Next, we have examined the present health policies in Ghana and their implications on cancer. We have critiqued their progress towards reducing the cancer burden and highlighted avenues and policy expansion. The existing policies assessed and critiqued include the non-communicable disease (NCD) policy, cancer control policy, public healthcare coverage, health technology assessment, traditional medicines policy and implementation of cancer policy on screening.

### Non-communicable disease policy

The National policy for non-communicable diseases (NCD) was formally adopted in Ghana in 2012 [74, 75]. This took a life course approach to focus on reducing prevalence and morbidity and increasing the quality of life for persons with NCDs [75]. The NCD Control and Prevention Programme (NCDCP), established by the Ministry of Health in 1992 was responsible for the implementation of a NCD strategic plan, which was developed by an expert steering committee. Given epidemiological evidence of an increase in obesity in Ghana [76], which is a risk factor for multiple cancers, primary NCD prevention strategies including diet and exercise could lead to a reduction in the cancer burden, as well as specific cancer screening and treatment policies.

However, in 2019, in-depth interviews with policy makers indicated it was challenging to implement the plan due to inadequate funding, poor intersectoral coordination and operationalisation of policies at subnational levels [74]. An international strategic dialogue on NCDs was held in Accra, in April 2022, with the World Health Organization, and Governments of Ghana and Norway. Here the Government of Ghana affirmed their commitment to tackle NCDs. The policy and strategic plans were both revised and published in 2021 for 2022–2026. In which key areas highlighted for addressing NCDs were reducing risk factors, making multisectoral action and taking a people-centred approach [77]. Building on the earlier strategy, it identifies that some progress has been made, such as the development of national cancer treatment guidelines, the inclusion of breast cancer in the NHIS package, and tightened tobacco legislation [78]. However, the lack of funding for the NCDCP and NCD-related activities, inadequate staffing, poor quality data and lack of coordination of the NCDCP across the country were barriers to achieving other objectives of the strategy thus far [78]. The revised approach involved multisectoral collaboration with the ministries responsible for security, education, gender and social protection [78]. The first objective of the plan focuses on primary prevention through health promotion, which may help to reduce risk factors associated with many NCDs including cancers [78]. The document also identifies *‘Objective 5: Ensure sustainable funding and other resources for NCD prevention and control*’, of which the second strategy was: ‘*Expand NHIS benefit package to include wellness services, childhood cancers and prostate cancer’* [78]. This indicates increasing political motivation to tackle many cancer-associated risk factors and make cancer treatment affordable. However, strategies to overcome the barriers identified, such as financing and coordination of the NCDCP, are not explicitly made clear and no organisations are identified as responsible for this within the implementation framework [78].

### Cancer control policy

The National Strategy for Cancer Control in Ghana (2012–2016) [3] was developed by the National Cancer Control Steering Committee (NCCSC) and was endorsed by the Minister of Health. It sets out targets to improve screening and detection, early diagnosis and treatment, palliative care and to establish a registry to monitor and respond to cancer trends [3].

The strategy acknowledges that one third of cancers in Ghana are preventable, one third can be treated with early diagnosis and adequate management, whilst those which are terminal would benefit from good care to increase their quality of life [3]. Through modifying lifestyle factors (for example diet, obesity, smoking, alcohol, occupational exposures) the policy aimed to decrease cancer cases by 30% [3]. It recommended adoption of the routine HPV vaccination of girls as part of a national programme and voluntary HBV vaccination in adults, as well as the use of legislation and taxes to reduce the consumption of unhealthy foods and beverages, and tobacco smoking [3].

Improved early detection was suggested by increasing awareness in pre-‘at-risk’ age categories as well as screening for cervical cancer and breast cancer (by clinical breast examination), with mammograms used as a diagnostic tool given the resource restraints [3].

Regarding treatment, this policy has set out an objective to improve effective diagnosis and treatment by 30% using evidence-based cost-effective interventions [3]. However, it notes financial barriers and increased funding are needed to realise this. Within the strategy it notes the psychosocial impact of cancer, which also affects family members and social relationships, and the plural health seeking behaviour of patients. To meet the treatment objectives, it recommends a multi-disciplinary team. It states treatment should be tailored to the patient and respect local culture [3]. Equitable access is a guiding principle of the strategy, however there are no plans to explain how this will be made affordable or integrated into the current NHIS.

Considering the care continuum, the strategy highlights the importance of improving quality of life and access to palliative care [3]. To measure progress towards these objectives, the strategy notes improved record keeping and documentation of cancer cases and establishing a national cancer registry will be vital.

Regarding implementation, the strategy highlights the role of the NCCSC to advise and assist the Ministry of Health in implementing the cancer strategy and integrating this with the current National Health Plan, as well as establishing the registry and advising on monitoring and evaluation to aid cancer prevention [3]. However, the progress on implementing the plan and meeting the 2012–2016 targets has not been reported at the time of writing. This may reflect similar challenges with funding, capacity and resource as suggested in the NCD plan [78, 79].

Although the strategy contains numerous promising policies and indicates political will to elevate the cancer burden, there is a lack of evidence on the implementation of the plan and a considerable gap remains in understanding the impact the strategy is having.

### Ghana National Health Insurance Scheme as a policy initiative

With the goal of achieving universal healthcare, the Ghana National Health Insurance Scheme (NHIS) was the first national health policy initiative in Africa, established in 2003 [30] and operational from 2005 [80]. The scheme is run by the Ghana National Health Insurance Authority, and their services are offered at both public and faith-based facilities. Enrolment in the scheme requires members to pay an annual premium/fee unless they fall into an exempt category (such as those requiring social protection, pregnant women, children and retirees). Those not enrolled are required to pay for treatment at point of care (out-of-pocket). The scheme is funded by the user levy, Value Added Tax (VAT) and Social Security and National Insurance Trust (SSNIT) deductions [30].

By 2014, 40% of the population were enrolled into the Scheme. However, with its increasing uptake, expenditure has out-paced the growth of the fund since 2009. By 2014, it reached a deficit of 300 million Ghana Cedis, [30] as stated in a World Bank report in 2017. They note that efforts are required to increase the financial sustainability of the scheme [30]. There have also been several challenges with the scheme including inequity in enrolment between income groups and delays in claim reimbursement to providers of the scheme [30, 81].

There has been considerable inequity in uptake of the NHIS, meaning many individuals risk catastrophic costs. Non-coverage has been found to be concentrated in poor, rural households with less formal education [80]. Although out of pocket spending has been found to decline, it remained at 62% in 2014 [82]. Reasons for non-enrolment are complex and multi-faceted, including poverty, traditional norms and experiences and confidence in the health system [83].

Two major cancers - breast and adult cervical - are covered by the NHIS [30]; this was extended to childhood cancers [84] in 2022 and reports suggest prostate cancer will be added in 2023 [85]. This is promising given the overwhelming financial burden of prostate cancer reported in 2022 [86]. However, despite breast and cervical cancers being covered by the scheme [30], patients still report catastrophic costs and the inability to afford treatment [12, 14]. This has both financial and psychological ramifications on patients and their families [13]. Moreover, it has been found that 58% or more of those requiring surgery (for both cancer and other conditions) face catastrophic costs even when enrolled on the scheme [87].

An assessment of cancer medicines pricing, availability and affordability in Ghana found average availability in public hospitals to be 14%, far below the WHO target of 80% [88], pushing patients to source medicines outside of hospitals. Moreover, numerous cancers remain uncovered by the scheme - treating colon cancer would require 2554 days wages (2022) [88].

Cancer can have devastating impacts on patients’ health, wellbeing and economic prosperity for themselves and their families, leading to impoverishment [89–91]. Maladaptive financial coping strategies incur debt and missed education by patients and their families. This can result in spiralling poverty, exacerbation of existing inequality and hindrance of social and economic progress in marginalised groups [91, 92].

Approaches to improve equity in uptake of the NHIS in Ghana in the most deprived and vulnerable groups have potential to decrease health inequalities. However, when considering cancer inequality in particular, this is required in tandem with expansion of the NHIS package to include more cancer treatments, and other costs associated with cancer care, such as those for diagnosis. Yet, governmental financing of cancer medicines is budget demanding. Many cancer medicines are high cost due both to the repercussions of intellectual property rights and the international trade environments [93] including for generic medicines [94].

### Health Technology Assessment (HTA) policy

Balancing the demand for increased cancer coverage poses a challenge in the face of the yearly deficit incurred by the NHIS. As there is a disproportionate amount of spending on medicines, thus health technology assessment (HTA) could be used to support more effective resource allocation [30].

Ghana is one of the first countries in Africa to formalise their HTA platform through work between Ministry of Health and the International Decision Support Initiative. The platform has recently gained pace. They now have a standardised process document and case studies [84, 95]. HTA for medicines has now been incorporated into the National Medicines Policy and the Ministry of Health are looking at ways to further adopt HTA across their current decision making processes [96]. Several case studies developed so far have suggested the initiative has potential to improve the cost-effectiveness of the medicines offered under the NHIS scheme [97].

Moreover, some cancer medicines have featured among Ghana’s HTA priorities. A HTA launch and report for childhood Burkitt’s Lymphoma treatment identified it as cost-effective in 2022. Following this, the Childhood Cancer Initiative with the NHIS was launched and four childhood cancers added to the NHIS package.

Although promising, a challenge remains in assessing the cost-effectiveness of cancer treatment in Ghana due to the lack of national cancer registry data. This is required to document cases and plan services based on projections of demand. The establishment of the Kumasi population registry means they now provide the first population based cancer estimates for that region [4]. Nevertheless, there is no current evidence of progress towards a national population cancer registry, despite its inclusion in the cancer control strategy [3].

Another future gap may be in resource needed to carry out all required HTAs. The approach so far has executed a small number of HTAs and implementing HTA more widely will require substantial capacity and resource allocation. Policy approaches to HTA involving external submission by companies (akin to the approach in Australia, Canada and Scotland) [98] may offer avenues to reduce the capacity burden.

### Other cancer policy initiatives: vaccination and screening programmes

In Ghana, there have been some independent efforts by charities and health facilities to initiate screening campaigns, both in breast cancer and cervical cancer, but to date no national programme exists. This is despite its inclusion in the cancer control strategy, which likely reflects the logistics and resource challenges highlighted previously.

There is strong evidence suggesting that both screening and prevention through HPV vaccination can reduce cervical cancer disease burden, and that these interventions are cost effective especially in low- and middle-income settings [99, 100]. This has underpinned global calls to eradicate the disease through such initiatives. With regard to breast cancer screening, the evidence is less clear [101]. A cost-effectiveness analysis of breast cancer screening in Ghana found that clinal breast examination would be cost-effective [102]. However, this assumes health workforce resource, competency and test accuracy seen in high income contexts, which may not be an accurate assumption. Other global guidelines have promoted governments firstly prioritising access to care [103]. The cost-effectiveness and feasibility of screening for prostate cancer is currently surrounded by ethical dilemma [104], particularly in low and middle income countries due to the cost and potential added burden of false positives.

. However, increased awareness could improve earlier diagnosis, as the national strategy promotes [3].

As previously noted, Ghana displays a high rates of liver cancer associated with HBV and HCV [23]. Implementation of HBV/HCV screening and vaccination, improving the knowledge of HBV/HCV status, and public awareness of the risks associated with this could improve earlier diagnosis of liver cancers. For cervical cancer, rollout of a nationwide screening and vaccination would be cost-effective at reducing the preventable cancer burden [105]. Alongside this, improving awareness of the signs, symptoms and risk factors of other cancers will also be important.

### Traditional medicines harmonisation policy

The final policy to be considered, relates to the previously described plurality of treatment and medicines and will show how several policies have attempted to harmonise, or even integrate, the traditional and orthodox sectors. The Ghana Ministry of Health established a Traditional and Alternative Medicine Directorate in April 2001 [44]. They have since launched a policy document [44]. In December 2021, the Recommended Herbal Medicine List and Basic Procedure for Assessing Efficacy and Safety of Herbal Medicine Products was also launched. The government has showed commitment to growth of traditional practices in the country through the Food and Drugs Authority approval of traditional medicines [106].

Since 2011, the government has run a pilot scheme integrating herbal medicines units to work side by side with biomedicine at 17 public hospitals [107]. These were in urban areas, although traditional medicines use is perceived as higher in rural regions [44, 107]. Despite calls, traditional medicines have not yet been included in the National Health Insurance Scheme (44,106).

Another challenge with traditional medicines has been the power dynamic between traditional and orthodox health care [38, 44]. Traditional medicines are often seen as unscientific [38]. However, several initiatives have sought to improve the scientific validity and reputation of traditional medicines [108]. This has been spearheaded by the Center for Scientific Research into Plant Medicine (CSRPM) established in Mampong-Akwapim [38]. In 2001, a bachelors’ degree in alternative medicines was introduced, seeking to improve the reputation and regulation of the use of herbal medicines [44]. Nevertheless, although traditional medicines broadly include a wide range of practices aiming to support physical, spiritual and social wellbeing, the policy initiatives so far are merely concerned with herbal medicines.

The lack of consideration of other forms of traditional care may relate to the requirement to adhere to the regulatory systems and evidence forms of biomedicine [38]. Some may also argue that many types of traditional medicines are based on different value systems and philosophies and involve interactions built of social experiences that cannot be clinically assessed in a trial fashion [38, 109].

Traditional medicines policy highlights an area where there have been advances in recent years. However, better understanding of the bottlenecks and barriers to delivery at a service level, such as through policy co-design and participatory approaches involving multiple stakeholders, would help this to be more effective. Kwame et al., conducted focus groups with a range of orthodox and traditional practitioners and service users, which highlighted how beliefs, positionality and power dynamics influence interactions between services [38]. Many of the traditional cancer treatments patients seek are also outside of the narrow focus on herbal medicines, so collaboration with a more diverse range of practitioners could also have mutual benefit to increase uptake of biomedical care.

## Conclusion and implications for future Ghana cancer treatment

This review has described the current but changing burden of cancer in Ghana and contextual factors which exacerbate it; five key factors have been argued to relate to this issue. Multiple policy changes have been identified but are argued to have key limitations in terms of their impact.

A key current omission is the availability of robust data, such as that provided through a national cancer registry; these are required to fully understand the disease burden and trends in Ghana. Such data will also be vital to accurately assess the impact of current and future policy initiatives and act accordingly. This, and many other objectives of the national cancer control strategy are a valuable starting point to curbing the cancer burden. In particular, interventions considered “best-buys” by WHO to prevent cervical cancer will be critical to implement [105]. At time of writing, there is little evidence of the cancer control strategy implementation signifying a need for greater prioritisation, financing, capacity building and human resource allocation to reduce cancer burden. Effective treatment pathways for cancer throughout the country, with sufficient staff education are key in resource-limited settings [103]. Improving cancer care in Ghana will require increasing the resource and capacity of healthcare staff to recognise, refer and treat cancer throughout the country, in particular, in poorer and more rural areas.

Multi-criteria decision making studies in Ghana suggest that decision makers also want to be able to consider the equity impacts of treatments [110]. At a recent stakeholder meeting, the ministry of health stated “leaving no-one behind” was an important premise of the SDG agenda, underscoring the importance of equity in their approach [84]. Critical are the associations between accessibility, outcomes, and inequalities with respect to cancer in Ghana, as marginalised and deprived groups are less likely to access cancer care due to belief systems and financial challenges, leading to poorer outcomes. Moreover, given the high patronage of traditional medicines, equitable approaches should further build collaboration with the sector. Thus, an equitable approach to cancer policy will need to incorporate the affordability of cancer medicines and harmonisation with community beliefs.

## Data Availability

Data sharing not applicable as this is a review of literature, and no datasets were generated.

## List of abbreviations

CHAG: Christian Health Association of Ghana
CSRPM: Center for Scientific Research into Plant Medicine
HBV: Hepatitis B Virus
HCV: Hepatitis C Virus
HIV: Human Immunodeficiency Virus
HPV: Human Papilloma Virus
HTA: Health Technology Assessment
NCCSC: National Cancer Control Steering Committee
NCD: Non-communicable disease
NCDCP: NCD Control and Prevention Programme
NHIS: National Health Insurance Scheme
SSNIT: Social security and national insurance trust
VAT: Value added tax
WHO: World Health Organization
